# SAF-A Forms a Complex with BRG1 and Both Components Are Required for RNA Polymerase II Mediated Transcription

**DOI:** 10.1371/journal.pone.0028049

**Published:** 2011-12-06

**Authors:** Dzeneta Vizlin-Hodzic, Rikard Runnberg, Jessica Ryme, Stina Simonsson, Tomas Simonsson

**Affiliations:** 1 Department of Medical Biochemistry and Cell Biology, Institute of Biomedicine, University of Gothenburg, Gothenburg, Sweden; 2 Department of Clinical Chemistry and Transfusion Medicine, Institute of Biomedicine, Sahlgrenska University Hospital, Gothenburg, Sweden; Texas A&M University, United States of America

## Abstract

**Background:**

Scaffold attachment factor A (SAF-A) participates in the regulation of gene expression by organizing chromatin into transcriptionally active domains and by interacting directly with RNA polymerase II.

**Methodology:**

Here we use co-localization, co-immunoprecipitation (co-IP) and *in situ* proximity ligation assay (PLA) to identify Brahma Related Gene 1 (BRG1), the ATP-driven motor of the human SWI-SNF chromatin remodeling complex, as another SAF-A interaction partner in mouse embryonic stem (mES) cells. We also employ RNA interference to investigate functional aspects of the SAF-A/BRG1 interaction.

**Principal Findings:**

We find that endogenous SAF-A protein interacts with endogenous BRG1 protein in mES cells, and that the interaction does not solely depend on the presence of mRNA. Moreover the interaction remains intact when cells are induced to differentiate. Functional analyses reveal that dual depletion of SAF-A and BRG1 abolishes global transcription by RNA polymerase II, while the nucleolar RNA polymerase I transcription machinery remains unaffected.

**Conclusions:**

We demonstrate that SAF-A interacts with BRG1 and that both components are required for RNA Polymerase II Mediated Transcription.

## Introduction

Transcription of nuclear genes is mediated by RNA polymerase II (Pol II) and proceeds through three main phases referred to as transcription initiation, transcription elongation and transcription termination [1]. Each of these phases is regulated in a variety of more or less gene specific ways to produce appropriate amounts of mRNA. Eukaryotic gene transcription is further controlled by reversible covalent histone modifications like methylation, phosphorylation or acetylation, which serve to alter local chromatin structure, and by ATP-dependent chromatin remodeling which serves to actively disrupt histone-DNA interactions [2].

SAF-A, also known as heterogeneous nuclear ribonucleoprotein U (hnRNP U), is an abundant nuclear protein that binds single- and double-stranded DNA [3], [4] and RNA [5], and organizes chromatin into functional gene domains [6]. It is important during early embryonic development and previous reports have indicated that SAF-A is involved in transcriptional regulation of specific genes due to its association with elements within the promoter regions of *apo-D*, *bmal1* and developmentally regulated genes *shh*, *klf2* and *oct4* [7], [8], [9], [10], [11] as well as with transcription factors such as the glucocorticoid hormone receptor, WT1, BRN4, SOX2 and OCT4 [10], [12], [13], [14]. SAF-A has also been demonstrated to interact directly with Pol II in HeLa cells [15], [16] and mES cells [10]. Thus SAF-A has been implicated in a variety of events related to transcriptional regulation of gene expression. In addition SAF-A has been reported to interact with epigenetic controllers of gene expression like CREB-binding protein (CBP) and P300/CBP associated factor (PCAF) [17], [18], and more recently SAF-A was shown to also interact with DNA methyltransferase DNMT1 [19].

In mammals the ATPase enzymatic activity of Brahma (BRM) and BRG1, also known as SWI/SNF related matrix associated actin dependent regulator of chromatin 4 (SMARCA4), provides the energy needed to remodel chromatin. The BRG1 protein preferentially associates with acetylated histones [20], [21], [22], [23] and was recently reported to help Pol II overcome nucleosomal barriers during transcription elongation *in vivo* [24]. The *brg1* gene is expressed in ES cells [25], and the encoded BRG1 protein associates with the nuclear matrix [26], is involved in chromatin loop formation [27], interacts with Pol II [28], regulates expression of pluripotency genes [29], and has been reported to be important for embryonic development [30], [31]. In the same way the *saf-A* gene is expressed in ES cells [10], and the encoded SAF-A protein associates with the nuclear envelope [6], is involved in chromatin loop formation [6], interacts with Pol II [10], [15], [16], and has been reported to be important for embryonic development [11], [32]. These similarities prompted us to explore the possibility that SAF-A and BRG1 in some way co-operate to regulate gene expression in ES cells, and if so, to investigate functional aspects of a possible SAF-A/BRG1 interaction.

## Results

### SAF-A interacts with BRG1 in mES cells

**Figure 1:**
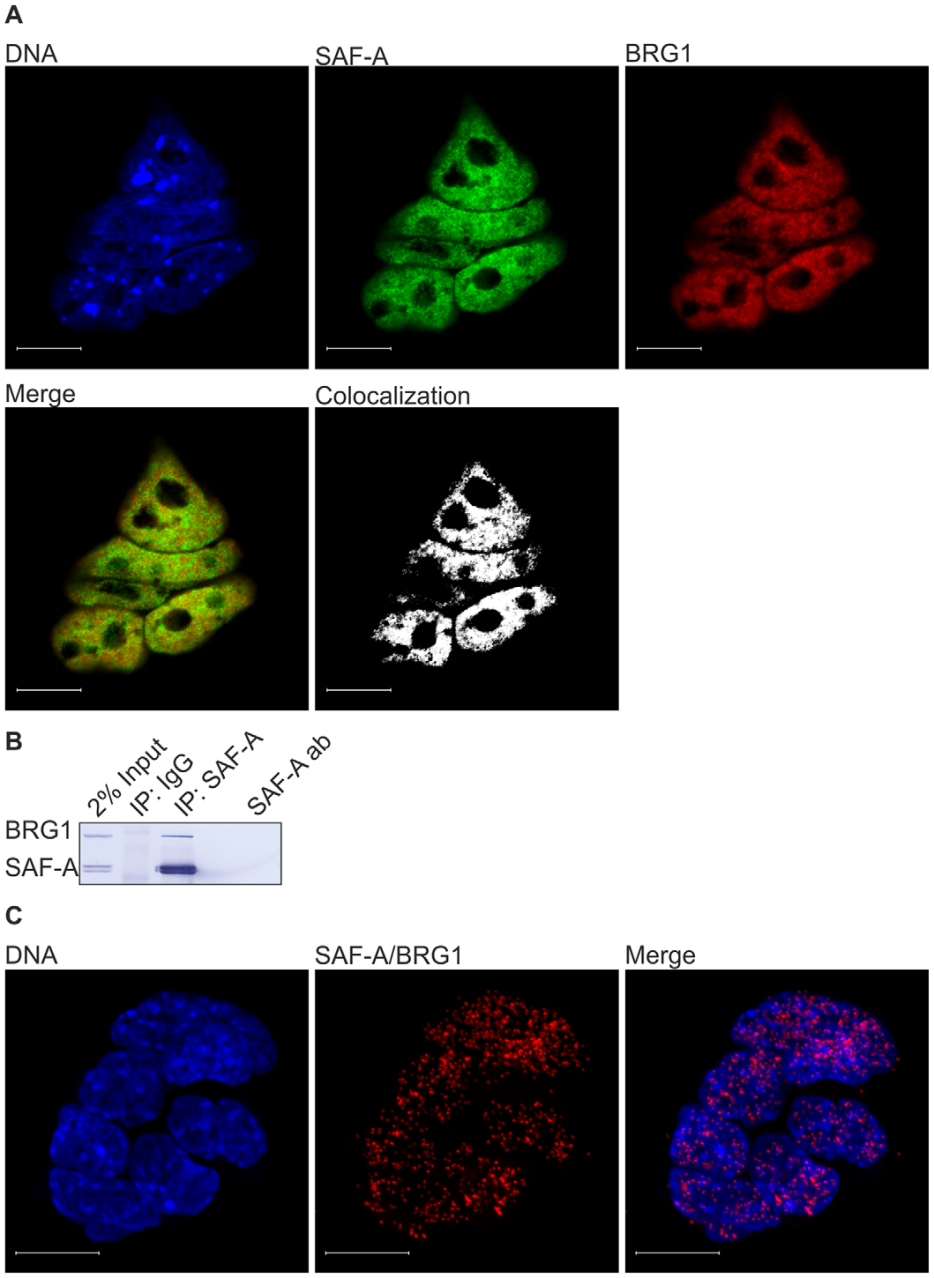
SAF-A and BRG1 interact in ES cells. (A) Immunoflurescent confocal microscopy shows that SAF-A (green) and BRG1 (red) localize to cell nuclei, and also reveals an almost complete co-localization between SAF-A and BRG1 (white). DNA was counterstained with DAPI (blue). Scale bars represent 10 µm. (B) Total ES cell extract was subjected to co-immunoprecipitation by anti-SAF-A and normal IgG as negative control. SAF-A-bound proteins were eluted, fractionated by SDS-PAGE and analyzed by Western blotting by anti-SAF-A and anti-BRG1. (C) Immunofluorescent confocal microscopy in combination with *in situ* proximity ligation assay was used to detect and visualize SAF-A/BRG1 interactions in ES cells. Irrespective of intensity each red dot represents a single endogenous SAF-A protein in close proximity to a single endogenous BRG1 protein. Confocal micrographs were collected at 0.38 µm intervals to create Z axis stacks, which were merged to render images of SAF-A/BRG1 interactions. DNA was counterstained with DAPI (blue). Scale bars represent 10 µm.

The spatial distributions of endogenous SAF-A and endogenous BRG1 were first examined in mES cells by immunofluorescence followed by confocal microscopy with sequential scanning of two channels, each corresponding to one protein. As expected, both SAF-A and BRG1 were found to localize to the nucleus of mES cells. The ImageJ software (http://rsb.info.nih.gov/ij), which was used to visualize the spatial distributions of endogenous SAF-A (Figure 1A, green) and endogenous BRG1 (Figure 1A, red) in interphase ES cells, reveals practically complete co-localization of SAF-A and BRG1 (Figure 1A, white).

The co-localization results encouraged us to perform co-immunoprecipitation (co-IP) experiments. Total extracts prepared from mES cells were subjected to co-IP with anti-SAF-A. SAF-A-immunoprecipitated proteins were fractionated by SDS- PAGE and analyzed by Western blotting using anti-SAF-A and anti-BRG1. Endogenous BRG1 was co-immunoprecipitated by anti-SAF-A whereas none of the analyzed proteins were co-precipitated by normal rabbit IgG (Figure 1B).

To confirm co-localization and co-IP results, and to visualize endogenous protein-protein interactions in single cells, we next employed *in situ* proximity ligation assay (PLA) [33]. Technical and biological controls (Figure S1) show that the PLA methodology is highly specific, and by using *in situ* PLA followed by confocal microscopy, a large number of endogenous SAF-A/BRG1 interactions can be visualized in nuclei of pluripotent mES cells (Figure 1C). This confirms the co-localization and co-IP results, and suggests that endogenous SAF-A interacts with endogenous BRG1 in mES cells.

### The SAF-A/BRG1 interaction does not solely depend on the presence of mRNA

**Figure 2:**
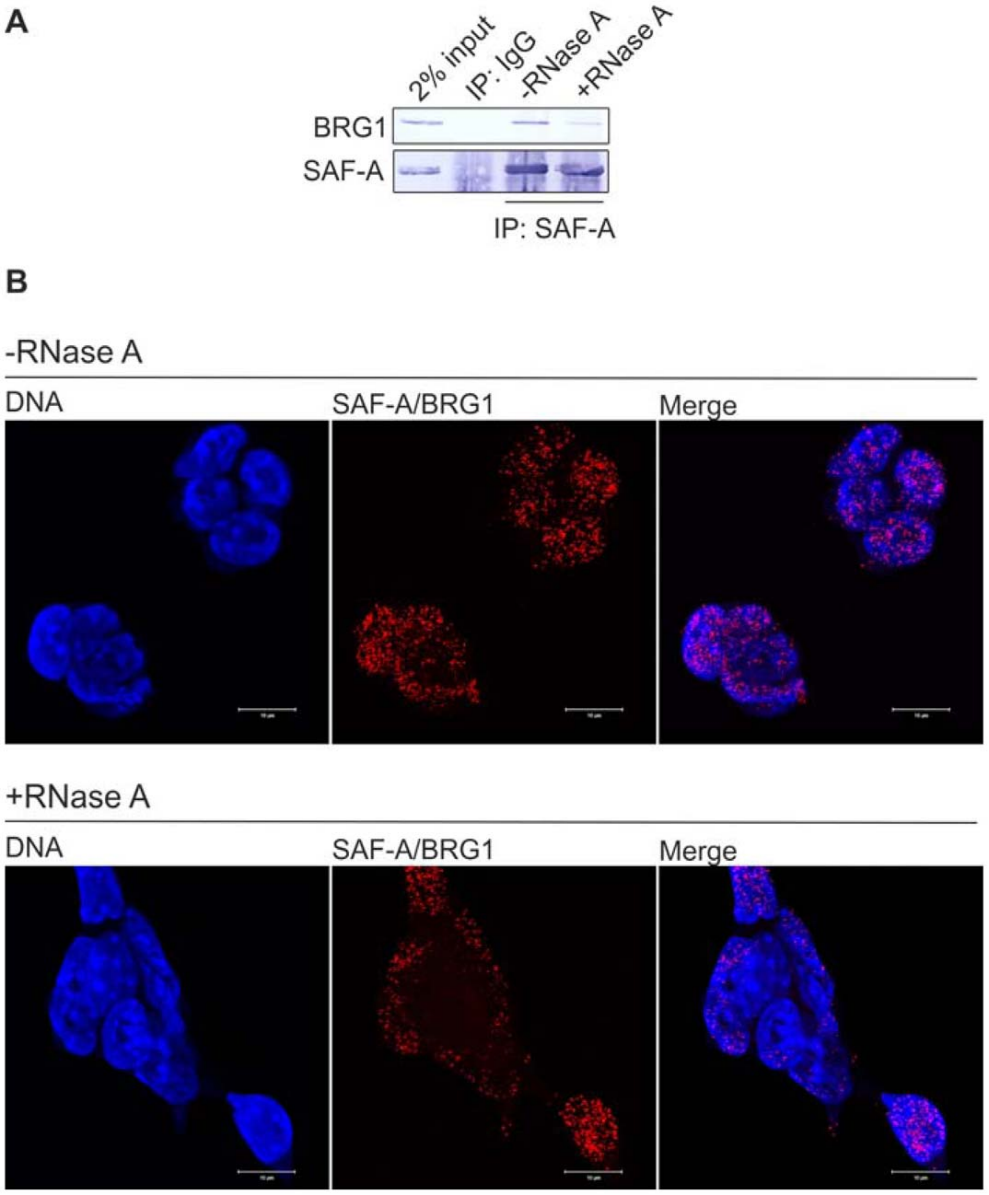
The SAF-A/BRG1 interaction does not solely depend on the presence of mRNA. (A) Untreated total ES cell extract, and total ES cell extract treated with RNase A, were subjected to co-immunoprecipitation by anti-SAF-A and normal IgG as negative control. SAF-A-bound proteins were eluted, fractionated by SDS-PAGE and analyzed by Western blotting by anti-SAF-A and anti-BRG1. (B) Immunofluorescent confocal microscopy in combination with *in situ* proximity ligation assay was used to detect and visualize endogeneous SAF-A/BRG1 interactions in untreated ES cells (-RNase A) and in ES cells treated with RNase A (+RNase A). Irrespective of intensity each red dot represents a single endogenous SAF-A protein in close proximity to a single endogenous BRG1 protein. Confocal micrographs were collected at 0.38 µm intervals to create Z axis stacks, which were merged to render images of SAF- A/BRG1 interactions. DNA was counterstained with DAPI (blue). Scale bars represent 10 µm.

Because hnRNP proteins such as SAF-A are involved in splicing [5], and the BRG1 homologue BRM has been implicated in splicing [34], we decided to investigate whether the SAF-A/BRG1 interaction depends on the presence of RNA. To this end total extracts prepared from mES cells were treated with RNase A followed by co-IP with anti-SAF-A. SAF-A-immunoprecipitated proteins were fractionated by SDS-PAGE and analyzed by Western blotting using anti-SAF-A and anti-BRG1 as above. This revealed that the SAF-A/BRG1 interaction does not solely depend on the presence of mRNA (Figure 2A). Similarly, using *in situ* PLA a large number of endogenous SAF-A/BRG1 interactions can be visualized in RNase A treated nuclei of pluripotent mES cells (Figure 2B). Thus given the comparably small size of RNase A (13.7 kDa) the SAF-A/BRG1 interaction demonstrated by Co-IP and PLA does not merely reflect that SAF-A and BRG1 are found in close proximity due to being simultaneously bound to the same mRNA molecule independently of each other.

In summary, RNase A treatment in combination with Co-IP and *in situ* PLA suggests that the SAF-A/BRG1 interaction does not solely depend on the presence of mRNA.

### The SAF-A/BRG1 interaction is unaffected by cellular differentiation

**Figure 3:**
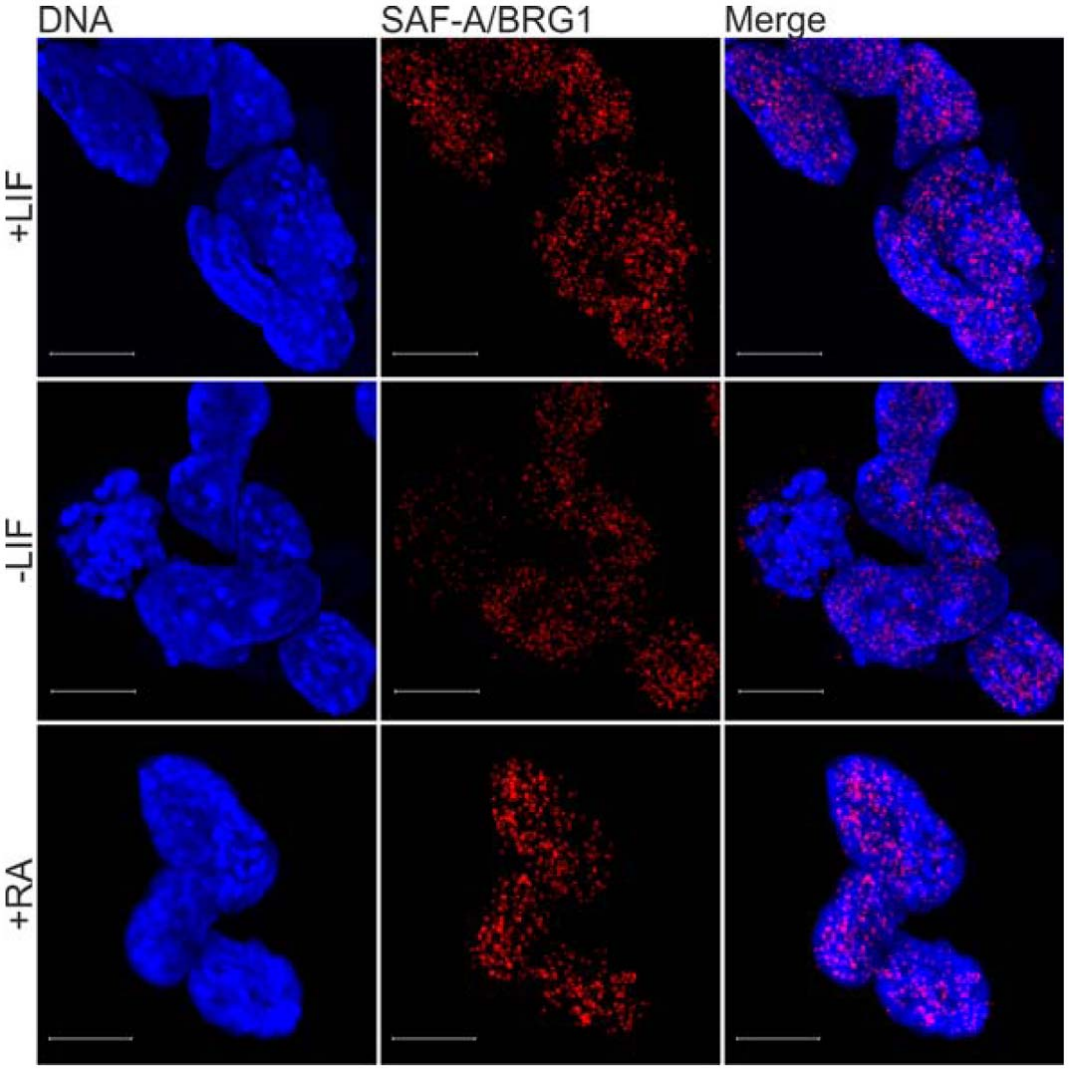
The SAF-A/BRG1 interaction is unaffected by cell differentiation. Immunofluorescent confocal microscopy in combination with *in situ* proximity ligation assay was used to detect and visualize endogeneous SAF-A/BRG1 interactions in ES cells and cells that were induced to differentiate by withdrawal of leukaemia inhibitory factor (−LIF) or by the addition of Retinoic Acid (+RA). Irrespective of intensity each red dot represents a single endogenous SAF-A protein in close proximity to a single endogenous BRG1 protein. Confocal micrographs were collected at 0.38 µm intervals to create Z axis stacks, which were merged to render images of SAF-A/BRG1 interactions. DNA was counterstained with DAPI (blue). Scale bars represent 10 µm.

To investigate if the observed SAF-A/BRG1 interaction is maintained during differentiation of mES cells, *in situ* PLA was performed and analyzed by confocal microscopy. Induction of mES cell differentiation was achieved by either withdrawal of leukemia inhibitory factor (LIF) (Figure S2, left panel), which is essential for pluripotency and self-renewal of mES cells [35], [36], or by the addition of RA to the culture medium (Figure S2, right panel). Cellular differentiation induced by either LIF withdrawal for two days (Figure 3, −LIF) or by the addition of RA (Figure 3, +RA) to the culture medium does not significantly affect the amount of endogenous SAF-A/BRG1 interactions in comparison to control experiments (Figure 3, +LIF). These findings suggest that the observed interaction between endogenous SAF-A and endogenous BRG1 is unaffected by cellular differentiation.

### SAF-A needs to interact with BRG1 for RNA polymerase II mediated transcription

**Figure 4:**
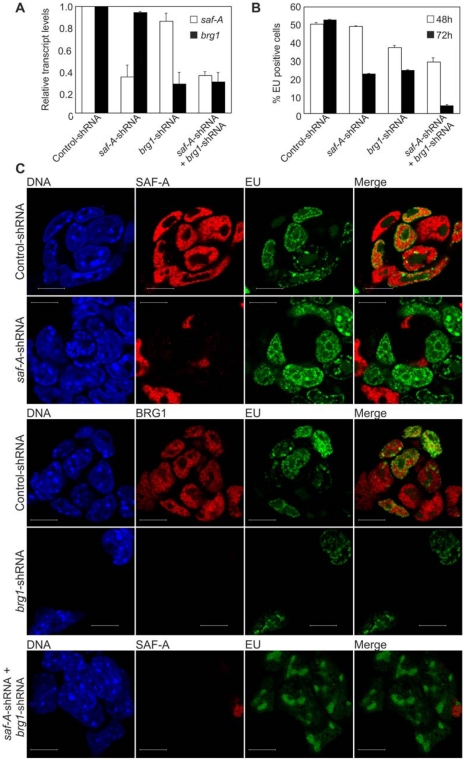
The SAF-A/BRG1 interaction is required for transcription by RNA polymerase II. (A) R1 mES cells were transfected with 0.8 µg of either control-shRNA or *saf-A*- and *brg1*-shRNA. Reverse transcriptase real time PCR analysis of control-, *saf-A*- and *brg1*-shRNA transfected ES cells under puromycin selection was performed 48 h post transfection. Expression levels are normalized to *gapdh*. Data are mean±SD (n = 3). (B) Global transcription was detected in control and knockdown mES cells by incorporation of 5-ethynyl uridine (EU) 48 h and 72 h post transfection. Percentage of EU positive knockdown cells in comparison to control cells 48 h (black bars) and 72 h (grey bars) post transfection. (C) EU incorporation (green) and endogenous SAF-A (red) or BRG1 (red) protein levels were analyzed by immonofluorescence confocal microscopy in control and knockdown mES cells 72 h post transfection. DNA was counterstained with DAPI (blue). Scale bars represent 10 µm.

We continued by exploring functional effects of SAF-A and BRG1. Knockdown of the *saf-A* and *brg1* genes on their own, as well as both genes together, was achieved by short hairpin (sh) RNA. In preliminary experiments four *saf-A*- and *brg1*-targeting constructs containing antibiotic selection were tested for their ability to knock down mRNA at three different concentrations 48 h post transfection. Similar effects were observed for all four independent *saf-A* and *brg1* targeting sequences. The two *saf- A*-shRNA and the two *brg1*-shRNA constructs that resulted in slightly more efficient knockdowns were used in subsequent experiments. At 48 h post transfection, *saf-A* and *brg1* transcript levels were lower in *saf-A*, *brg1* and in doubly *saf-A/brg1* depleted cells (Figure 4A). Albeit at 96 h post transfection, *saf-A* and *brg1* deficiency also yielded lower levels of total RNA (data not shown) as well as housekeeping gene mRNA. Western blotting confirms that shRNA generates a concentration dependent decrease of SAF-A protein 48 hours post transfection (Figure S3), even though SAF-A is known to be a very stable protein.

This discovery encouraged us to investigate whether deficiency of SAF-A, BRG1, or both proteins jointly, affects global transcription in mES cells. Global transcription was measured in live control, *saf-A*, *brg1* and *saf-A/brg1* knockdown mES cells by incorporation of 5-ethynyl uridine (EU) into nascent RNA transcripts under experimental conditions that reveal specific transcription by Pol II. Endogenous protein levels and EU incorporation was analysed by immunofluorescence confocal microscopy at two different time points post transfection. At 48 h post transfection, the knockdown cells were positive for EU incorporation although visible levels of the endogenous proteins were undetectable. However, quantification of *saf-A* and *brg1* knockdown effects on global transcription revealed that the proportion of transcriptionally active (EU positive) cells when SAF-A and BRG1 were depleted decreased by 1% and 26% in comparison to the negative control-shRNA, respectively. The dual SAF-A and BRG1 depletion resulted in a decrease of EU positive cells by 42% (Figure 4B). At 72 hours post transfection, endogenous SAF-A and BRG1 were undetectable (Figure 4C). The proportion of transcriptionally active (EU positive) cells when SAF-A and BRG1 were depleted decreased by 57% and 54%, respectively, in comparison to the negative control-shRNA. More notably, simultaneous depletion of SAF-A and BRG1 resulted in a dramatic 92% decrease of EU positive cells in comparison to the negative control-shRNA (Figure 4B, C). Albeit incorporation of EU in nucleoli could still be observed (Figure 4C). This indicates that RNA polymerase I mediated transcription is unaffected by depletion of SAF-A and BRG1, and points toward a specific and fundamental role for the SAF-A/BRG1 interaction in global Pol II mediated transcription.

## Discussion

In summary, we demonstrate by two independent techniques, co-IP and *in situ* PLA, that SAF-A, a constituent of the nuclear matrix, interacts with BRG1, the ATP-driven motor of the human SWI-SNF chromatin remodeling complex in mES cells, and that the interaction does not solely depend on the presence of mRNA. We also show that the SAF-A/BRG1 interaction persists when mES cells are induced to differentiate. In addition, shRNA mediated *saf-A* and *brg1* knockdowns in combination with EU incorporation analysis reveal that the SAF-A/BRG1 interaction is required for global Pol II mediated transcription, and also indicates that the proteins have complementary roles. Previous reports have demonstrated that both *saf-A* and *brg1* are expressed in ES cells [10], [25], and also that both encoded proteins associate with the nuclear matrix [6], [26], are involved in chromatin loop formation [6], [27], interact with Pol II [10], [15], [16], [28], regulate expression of pluripotency genes [10], [29] and are important for embryonic development [11], [30], [31], [32].

Moreover, both SAF-A and BRG1 have been reported to interact with actin [16], [37] and DNA topoisomerase IIβ [38], [39]. In combination with these reports, and the observation that transcription is preferentially regulated at the nuclear periphery in embryonic stem cells [40], our discoveries allow us to speculate about possible roles for a SAF-A/BRG1 complex in gene activation. First, a SAF-A/BRG1 complex may help activating a gene locus by transporting the relevant chromatin domain along the nuclear matrix until it reaches a transcription machinery, perhaps located in the vicinity of a nuclear pore. Second, when the relevant gene locus reaches the transcription machinery a SAF-A/BRG1 complex may help the formation of a chromatin loop, in which DNA topoisomerase IIβ helps Pol II overcome topological constraints during transcription.

Finally, SAF-A has been shown to decrease the rate of Pol II mediated transcription [15] and BRG1 has been demonstrated to increase the rate of Pol II mediated transcription [27], so once transcription has begun SAF-A and BRG1 may provide a brake and throttle, respectively, for Pol II during elongation. The activation of a specific gene locus obviously depends on a large number of additional factors, but it is worth noting that SAF-A, BRG1, Pol II, DNA topoisomerase IIβ and actin all are present in all cell types so far investigated by the human protein atlas project (www.proteinatlas.org). In conclusion, and regardless of exactly how a gene locus becomes transcriptionally active, the data presented here suggests that a SAF-A/BRG1 interaction is required for RNA polymerase II mediated transcription.

## Materials and Methods

### Cell cultures

Cell lines were grown at +37°C in humidified atmosphere containing 5% CO_2_. Murine ES cell line R1 [41] (derived from 129X1×129S1) was maintained on mitotically inactivated mouse embryonic fibroblast (MEF) feeder layers in Dulbecco's modified Eagle medium supplemented with 1 mM sodium pyruvate, 0.1 mM non-essential amino acids, 2.0 mM L-glutamine, 0.1 mM beta-mercaptoethanol, 15% foetal bovine serum, 20 mM HEPES (pH 7.3), 100 U/100 µg penicillin/streptomycin and 1000 U/ml LIF (ESGRO, Chemicon). Early differentiation of mES cells was established by either leukemia inhibitory factor (LIF) withdrawal or by the addition of retinoic acid (RA) from/to the culture medium for 1–4 days.

### Co-immunoprecipitations

Lysates were prepared from mES cells grown to 80–90% confluence. Cells were washed three times with PBS and 5 mM DTBP was added to stabilize protein-protein interactions prior to lysis in 50 mM Tris-HCl, pH 7.5, 0.15 M NaCl, 1% Triton X-100, 5 mM EDTA, 1 mM phenylmethyl sulphonyl fluoride, 10 µg/µl aprotinin, 1 µg/ml leupeptin and pepstatin A. Lysed cells were centrifuged for 10 min at 13,000×g. RNase A (Roche) was added to a final concentration of 0.1 µg/µl and incubated for 30 min where indicated. The supernatant was pre-cleared and incubated with antibodies and Protein A/G PLUS-Agarose beads (Santa Cruz) overnight at +4°C. Anti-SAF-A and anti-BRG1 conjugated beads were centrifuged at 1,000×g and the supernatant was discarded. The beads were washed three times with 1 ml of cold lysis buffer, resuspended in 20 µl 2×Laemli buffer, heated to +95°C for 3 min and centrifuged for 1 min at 1,000×g. Supernatants were collected and used for Western blot analysis.

### Western blotting

Proteins were separated using SDS-PAGE, electrotransferred onto polyvinylidene difluoride (PVDF) membranes for 1 h, 110 mA/gel in transfer buffer (48 mM Tris, 39 mM Glycin, 1.3 mM SDS, 10% MeOH) and immunologically detected. PVDF membranes were blocked with 5% non-fat dry milk/0.1% Tween 20/PBS (PBST) and incubated overnight with primary antibodies (anti-SAF-A (1∶1000; ab20666; Abcam), anti-BRG1 (1∶1000; sc-17796; Santa Cruz)) diluted in blocking solution. After washing with PBST, blots were incubated for 1 h with secondary antibodies (AP conjugated goat anti-mouse IgM+IgG+IgA (H+L) and AP conjugated goat anti-rabbit IgM+IgG (H+L chain specific) (1∶1000, SouthernBiotech)). Proteins were visualized with NBT/BCIP (Promega).

### Immunostaining

For imunofluorescence, cells were trypsinized, counted and 4.0×10^4^ cells were re- plated onto glass cover slips in 24-well dishes and cultured overnight. Cells were fixed with 4.0% paraformaldehyde/PBS for 20 min, washed, permeabilized with 0.25% Triton X-100/PBS for 5 min, blocked in 0.1% Triton X-100/10% FCS/PBS for 20 min. Primary anti-SAF-A (1∶500, Abcam) and anti-BRG1 (Santa Cruz; sc-17796; 1∶200) and secondary antibodies (Alexa Fluor 488 conjugated goat anti-rabbit IgG and Alexa Fluor 555 conjugated goat anti-mouse IgG (1∶500, Invitrogen)) were diluted in 0.1% Triton X-100/1% FCS/PBS and added for 2 and 1 h respectively, each followed by washes in 0.1% Triton X-100/PBS. Nuclei were counterstained with DAPI. Cover slips were air dried, mounted and analyzed on an inverted Zeiss LSM 510 META confocal microscope equipped with a Zeiss image processing system, using an 63×/1.4 oil objective and sequential scanning with the appropriate filter.

### *In situ* proximity ligation assay

3–4×10^4^ cells were grown on eight-well chamber slides overnight. Duolink (Olink Bioscience) *in situ* PLA was performed according to the manufacturer's protocol. Fixation, permeabilization, blockage and primary antibody incubations were performed as described for immunostaining analyzes. PLA probes were diluted in 0.1% Triton X-100/1% FCS/PBS and incubated in preheated humidity chamber for 1 h at +37°C, followed by hybridization, ligation, amplification and detection according to the manufacturer's protocol. Slides were analyzed on an inverted Zeiss LSM 510 META confocal microscope equipped with a Zeiss image processing system, using a 63×/1.4 oil objective and sequential scanning with the appropriate filter.

### ShRNAs and transfection

Four SureSilencing™ shRNA plasmids with *saf-A* (*saf-A*-1: ACA GTG TCT TGG CAA GTT TAT; *saf-A*-2: ACA GTG GTT TGT CTT GAT ACT; *saf-A*-3: AGG CCG TGG AGG ATT CAA TAT; *saf-A*-4: GAA CTC TCT TAT GCG AAG AAT) and *brg1* (*brg1*-1: CTC GAG GGT TCC CTG ATC TAT; *brg1*-2: TGA ACC TGG CTC TGA GTA TTT; *brg1*-3: GAC CAC CTA TGA ATA TAT CAT; *brg1*-4: GCC AAG GAC TTC AGG GAG TAT) specific insert sequences were obtained from SABiosciences. Preparation of shRNA was done as recommended by the manufacturer. 5×10^4^ ES cells were transfected 4 h post seeding with 0.4, 0.8 and 1.0 µg shRNA complexed with Lipofectamine™ LTX Reagent (Invitrogen) according to the manufacturer's instructions. For each concentration of shRNA, an equivalent concentration of negative control-shRNA was used. Transfected cells were selected by addition of puromycin to a final concentration of 1 µg/ml (Calbiochem) 24 h post transfection. 48 and 96 h post transfection cDNA levels of *saf-A*, *brg1* and *gapdh* were detected by real time PCR. Global transcription status of the ES cells was analyzed 48 and 72 h post transfection using immunofluorescence confocal microscopy.

### RNA preparation/reverse transcription/real time PCR analysis

Total RNA was extracted using RNeasy minikit (Qiagen). Contaminating genomic DNA was eliminated with RNase-Free DNase (Qiagen). cDNA synthesis was performed with SuperScript III kit (Invitrogen). Endogenous mRNA levels were measured by RT-qPCR analysis based on SYBR Green detection. Briefly, the RT-qPCR mixture contained 1 µl of the reverse transcription reaction product in a total volume of 20 µl, containing 1×SYBR Green mix reagent (Applied Biosystems), 50 nM forward primer and 50 nM reverse primer. Each sample was analyzed in duplicate using the following oligonucleotide pairs: *saf-A* Fwd (GCC GAG GGT ATT TTG AGT ACA T) and *saf-A* Rev (TGT GTC ATC GAA GTG TTC GTC TT); *gadph* Fwd (AAT GTG TCC GTC GTG GAT CTG A) and *gadph* Rev (GAT GCC TGC TTC ACC ACC TTC T). All primer pairs yielded a single product as confirmed by dissociation curve analyses, and gave no product in the no-template control.

### Global transcription assay

Global transcription was examined using Click-iT® RNA Imaging Kit (Invitrogen). 5- ethynyl uridine (EU) was added at a final concentration of 1 mM and incubated under normal cell culture conditions for 1 h followed by fixation in 4% paraformaldehyde/PBS for 20 min and permeabilized with 0.25% Triton X-100/PBS for 10 min. EU incorporation was detected according to the manufacturer's protocol with the optional antibody detection step included. Primary anti-SAF-A (1∶500, Abcam), anti-BRG1 (1∶200, Santa Cruz) and secondary antibody (Alexa Fluor 555 conjugated goat anti-mouse IgG (1∶500, Invitrogen)) were diluted in 0.1% Triton X-100/1% FCS/PBS and added for 2 and 1 h respectively, each followed by washes in 0.1% Triton X-100/PBS. Nuclei were counterstained with 4,6-diamidino-2-phenylidole (DAPI). Fixated cells were analyzed on an inverted Zeiss LSM 510 META confocal microscope equipped with a Zeiss image processing system, using an 63×/1.4 oil objective and sequential scanning with appropriate filters.

## Supporting Information

Figure S1 ***In situ*** **PLA is highly specific.** The omission of one or both primary antibodies yields no detectable signal (negative technical controls). As a positive biological control the previously reported interaction between SAF-A and Pol II is clearly visualized by *in situ* PLA. DNA was counterstained with Hoechst 33342 (blue). Scale bars represent 10 µm.
(TIF)

Figure S2 **Transcript levels of** ***saf-A*** **and** ***brg1*** **correlate during the differentiation of murine embryonic stem cells.** Transcript levels of *saf-A*, *brg1* and *oct4* were determined in undifferentiated and mES cells that were induced to differentiate either by withdrawal of leukemia inhibitory factor (-LIF, left panel) or by the addition of retinoic acid (+RA, right panel). Samples were collected at twenty-four hour intervals for four days following LIF withdrawal or RA treatment, respectively. Samples were analyzed by real time PCR, and transcript levels were normalized to *gapdh* mRNA levels. Data are mean±SD (n = 3).
(TIF)

Figure S3 **Western blotting confirms that shRNA generates a concentration dependent decrease of SAF-A protein level 48 hours post transfection.**
(TIF)
