# Supplementary figures and images for: SAF-A Forms a Complex with BRG1 and Both Components Are Required for RNA Polymerase II Mediated Transcription
Source: PLoS One. 2011 Dec 6;6(12):e28049. doi: 10.1371/journal.pone.0028049 (PMC3232189; doi:10.1371/journal.pone.0028049)

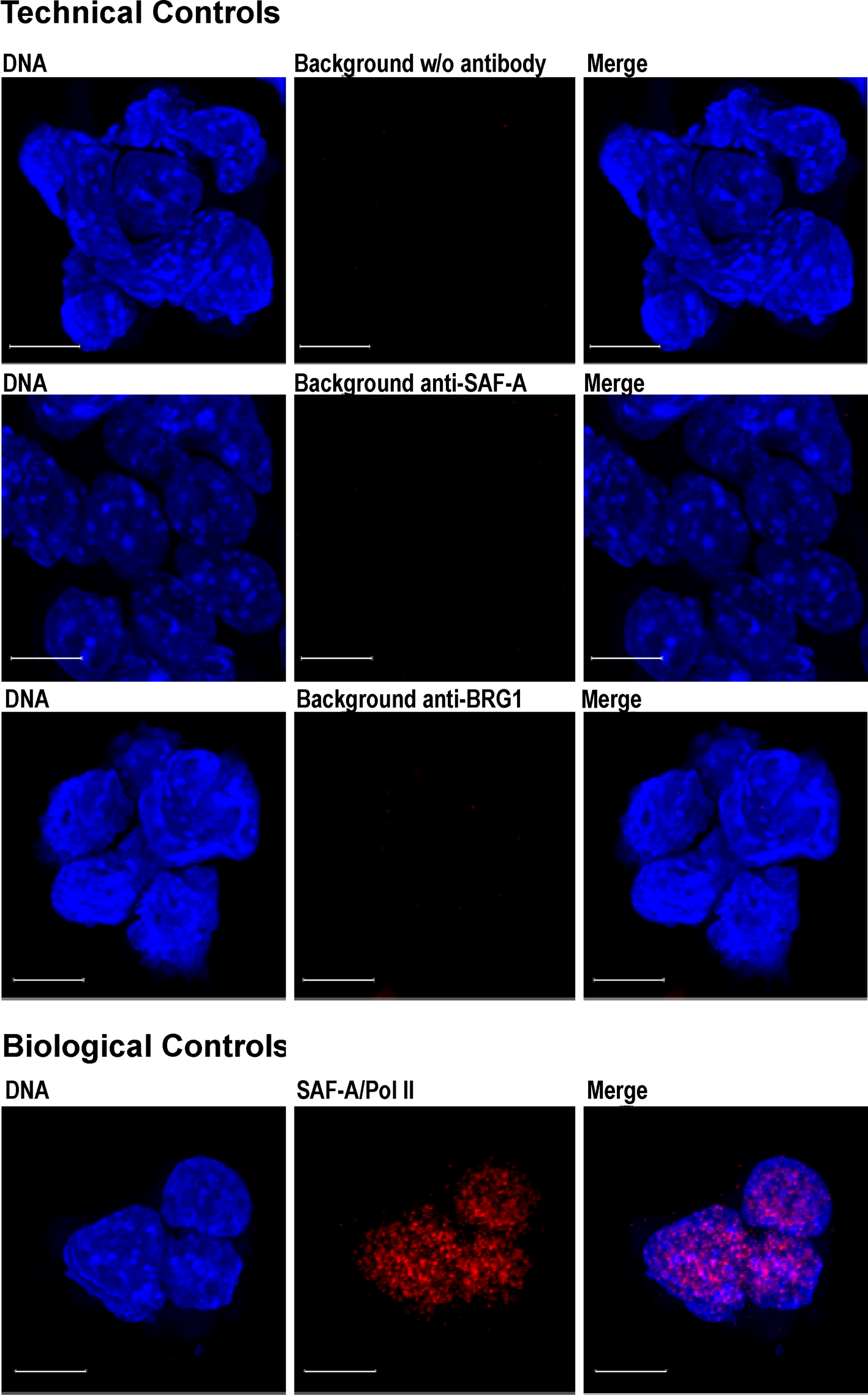

Supplement: Figure S1 — In situ PLA is highly specific. The omission of one or both primary antibodies yields no detectable signal (negative technical controls). As a positive biological control the previously reported interaction between SAF-A and Pol II is clearly visualized by in situ PLA. DNA was counterstained with Hoechst 33342 (blue). Scale bars represent 10 µm. (TIF) [file pone.0028049.s001.tif]

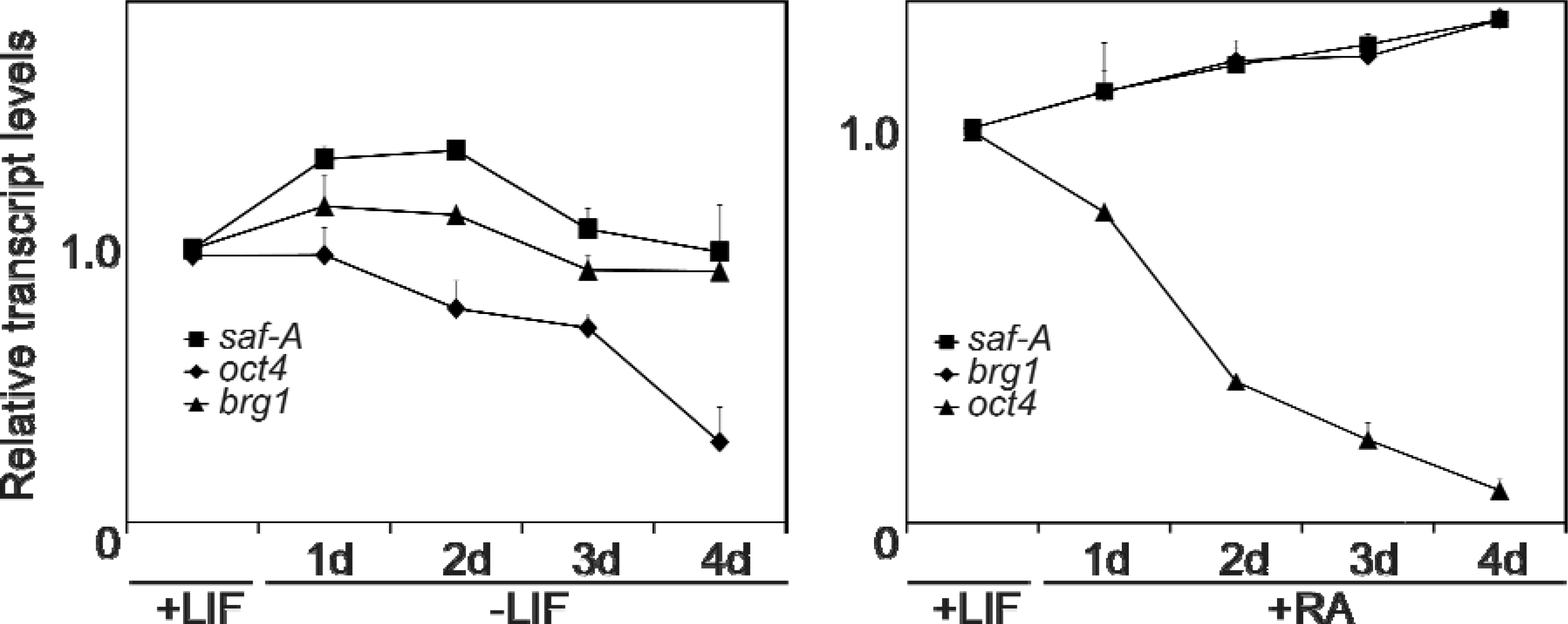

Supplement: Figure S2 — Transcript levels of saf-A and brg1 correlate during the differentiation of murine embryonic stem cells. Transcript levels of saf-A, brg1 and oct4 were determined in undifferentiated and mES cells that were induced to differentiate either by withdrawal of leukemia inhibitory factor (-LIF, left panel) or by the addition of retinoic acid (+RA, right panel). Samples were collected at twenty-four hour intervals for four days following LIF withdrawal or RA treatment, respectively. Samples were analyzed by real time PCR, and transcript levels were normalized to gapdh mRNA levels. Data are mean±SD (n = 3). (TIF) [file pone.0028049.s002.tif]

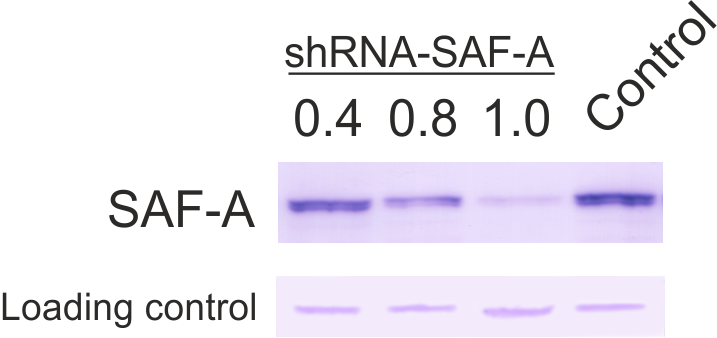

Supplement: Figure S3 — Western blotting confirms that shRNA generates a concentration dependent decrease of SAF-A protein level 48 hours post transfection. (TIF) [file pone.0028049.s003.tif]
